# Stochasticity in cultural evolution: a revolution yet to happen

**DOI:** 10.1007/s40656-017-0173-y

**Published:** 2017-11-27

**Authors:** Sylvain Billiard, Alexandra Alvergne

**Affiliations:** 1 Univ. Lille, CNRS, UMR 8198, Evo-Eco-Paleo, 59000 Lille, France; 2 School of Anthropology and Museum Ethnography, University of Oxford, Oxford, UK

**Keywords:** Cultural evolution, Anthropology, Archaeology, Stochastic processes, Population genetics, Population ecology

## Abstract

Over the last 40 years or so, there has been an explosion of cultural evolution research in anthropology and archaeology. In each discipline, cultural evolutionists investigate how interactions between individuals translate into group level patterns, with the aim of explaining the diachronic dynamics and diversity of cultural traits. However, while much attention has been given to deterministic processes (e.g. cultural transmission biases), we contend that current evolutionary accounts of cultural change are limited because they do not adopt a systematic stochastic approach (i.e. accounting for the role of chance). First, we show that, in contrast with the intense debates in ecology and population genetics, the importance of stochasticity in evolutionary processes has generated little discussion in the sciences of cultural evolution to date. Second, we speculate on the reasons, both ideological and methodological, why that should be so. Third, we highlight the inadequacy of genetically-inspired stochastic models in the context of cultural evolution modelling, and ask which fundamental stochastic processes might be more relevant to take up. We conclude that the field of cultural evolution would benefit from a stochastic revolution. For that to occur, stochastic models ought to be developed specifically for cultural data and not through a copy-pasting of neutral models from population genetics or ecology.

## Introduction

Evolutionary theory has been applied to the study of culture in various ways for more than 100 years. In the field of cultural evolution, most, if not all, of the approaches developed until the 1970s were narrative-based and interpretive, that is, there were no quantitative predictions for how cultural traits (e.g. behaviours, ideas, artefacts) should vary and be distributed in a population. Contemporary cultural evolution research differs markedly from these previous traditional approaches in that it is built upon a quantitative and mathematical framework. Building on precursors like Gulick (1905) and Binford (1963), a quantitative framework was developed in 1981 by two evolutionary scientists, Cavalli-Sforza and Feldman (1981). They borrowed mathematical tools from population genetics to predict how the social transmission of information between individuals influences the dynamics of culture at the population level. Cavalli-Sforza and Feldman (1981) contributed two main concepts: (1) cultural selection, analogous to natural selection, which describes the differential reproductive success of cultural traits, considered as deterministic, and (2) cultural drift, analogous to genetic drift, which accounts for the role of chance in cultural change.

Following Cavalli-Sforza and Feldman’s seminal work, there has been an explosion of cultural evolution studies in anthropology and archaeology (reviewed in Mesoudi 2011; Lewens 2015), mostly focusing on social transmission mechanisms, for instance the importance of vertical social transmission (i.e. from parents to offspring) as compared with horizontal social transmission (i.e. from peers). Subsequently, the anthropologists Boyd and Richerson (1985) and their students extended Cavalli-Sforza and Feldman’s framework to include various modes of cultural selection, which they coined biased cultural transmission, e.g. prestige-bias and conformist-bias but also payoff-biased social transmission (Henrich and McElreath 2003). Overall, the concept of cultural selection, though arguably more complex than the concept of natural selection, fell on fertile soil in the human sciences as testified to by the intense debates between the schools of cultural (Acerbi and Mesoudi 2015) and cognitive anthropology (Claidière et al. 2014) over what cultural selection actually entails (see also Lewens 2015 for a dispassionate review).

Comparatively to the success encountered by the concept of cultural selection, the concept of cultural drift has been much less popular than that of cultural selection, and it remains little explored. There has undoubtedly been a productive utilisation of neutral models in archaeology and anthropology (neutral models are a special category of stochastic models where population change is not pushed towards a particular direction, “Appendix 1”). To date, however, there have been little controversies over the role of selection versus drift for understanding the evolution of culture and patterns of cultural diversity. This is in sharp contrast with the biological sciences, within which the relative importance of selection and drift for explaining both species diversity and evolutionary change has been intensely debated for years. In population genetics and ecology, it led to a stochastic revolution whereby the cause of change by default is not assumed to be selection anymore but rather stochastic processes, in particular genetic drift and demographic stochasticity (Kimura 1983; Hubbell 2001). Broadly speaking, selection models must now provide more explanatory power than neutral models (models without selection) for the data at hand to be accounted for by selection.

In the field of cultural evolution, such a stochastic revolution has yet to happen. Questioning the role of stochasticity for explaining patterns of cultural diversity is necessary, however, for several reasons. First, there is strong empirical evidence that there is a large population-level variance or noise around the mean value of cultural traits, which is neglected by classic deterministic approaches. Given social systems are subject to stochastic effects, properly defining and modelling random fluctuations around the population mean of cultural traits is key for determining the extent to which cultural diversity is underpinned by adaptive processes. Second, since agents of cultural evolution are discrete (individuals) and populations are finite, stochasticity is necessary to explain some features of the diachronic dynamics of cultural traits. For instance, fluctuations and the extinction of a trait cannot be modelled with classical approaches based on differential equations. Third, stochasticity is a fundamental concept in psychology and neurobiology for making sense of how individuals make decisions (Forstmann et al. 2016). Fourth, in contrast with evolutionary biology and ecology (where the Wright-Fisher’s and Moran’s models are classically used), there is no consensus over which stochastic process is the referent one for cultural evolution models. Finally, since random genetic drift and natural selection are equally important in the neo-Darwinian theory of evolution, the role of stochasticity in driving cultural change and diversity must be considered to adequately evaluate the relevance of the analogy between biological and cultural evolution.

In this paper, our overarching goal is to evaluate the need for a stochastic revolution in the field of cultural evolution by contrasting the uses and utility of stochastic models in the biological sciences (i.e. population genetics and ecology) and in some of the human sciences engaging with cultural evolution research (i.e. archaeology and anthropology). First, we discuss the role given to stochasticity in the cultural evolution literature. Second, we question why there has only been a few controversies over the role of stochasticity in cultural evolution as compared with the intense debates that neutral theory generated in ecology and population genetics. Finally, we dispute the analogy between cultural and genetic drift and its relevance for the study of cultural evolution. We conclude that a stochastic revolution is much needed in contemporary cultural evolution studies, albeit not in a copy-paste fashion from the biological sciences, but after the sources of stochasticity unique to human culture have been identified. Such a paradigm change from a deterministic to a stochastic view of the world has proven to be fruitful in several scientific disciplines including physics, chemistry, biology and psychology (Gigerenzer et al. 1989; Hacking 1990). We contend that it would also be productive for advancing the field of cultural evolution because making chance a central concept will allow a better description of the processes underpinning cultural evolution and an increased control of uncertainty when interpreting observations.

## The role of stochasticity in the evolution of culture

In this section, we give an historical overview of the concept of cultural drift in archaeology and anthropology. We begin by showing how cultural drift is rooted in the concept of chance, which has multiple meanings and uses in the sciences of cultural evolution. We then outline how the concept has been mathematically formalised and how it is used to interpret archaeological and anthropological data.

### The multiple meanings and uses of the concept of chance in cultural evolution studies

The concepts of chance, randomness or probability have ubiquitous meanings and interpretations, partly because of their uses for various purposes in both science and philosophy (see Hájek 2012 for an extensive review of all interpretations of probability). This is of course true of the social sciences, especially in cultural evolution studies in which two main interpretations are commonly used: subjective and objective probability (Hacking 1990). Both interpretations of probability appeared early in the history of the sciences of cultural evolution, as outlined below.

#### Subjective probability

Subjective probability (sometimes also called Bayesian probability) is a measure of the degree of confidence of an explanation or a prediction, given some necessarily limited information. The concept assumes that given a set of known facts about the roll of a dice, the confidence associated with a result can be measured. Subjective probability can be used to produce statistical tests, which facilitate the analysis and the interpretation of data by controlling for a degree of uncertainty. Subjective probability can even be applied to the deterministic processes driving cultural evolution, for instance, when knowledge of the initial conditions is limited. It is this interpretation of probability that is often implied in the early anthropological accounts of cultural change, for instance those provided by Franz Boas and Claude Lévi-Strauss. Boas argued that the use of probability distributions was necessary to describe and analyse the evolution of humans and cultures. Indeed, all individuals have their own experience and history, which make them unique because ‘There are so many uncontrollable conditions that influence the development of the organism that even with identical ancestry the same form and size cannot always be expected.’ (Boas 1938, p. 39). Lévi-Strauss (1952, p. 61) used similar arguments ‘To explain differences in how civilisations unfold, we would have to invoke sets of causes so complex and discontinuous that they would not be knowable. This justifies the introduction of the notion of probability in the social sciences.’^1^ (our translation). Throughout his book, Lévi-Strauss uses chance to justify metaphorically that some civilisations appear more advanced than others: it is not because of their intrinsic differences but because of their history that some civilisations accumulated more technological and cultural innovations (it is worth noting here that Lévi-Strauss falls into the classic trap of conflating chance with contingency). Even though both Boas and Lévi-Strauss were inclined to use the concept of chance, they both had a deterministic view of the processes underlying cultural evolution. Boas for instance wrote that “If we could control all the conditions […] and if we could make all of these uniform, then we should, of course, expect the same result in every case”. (Boas 1938, p. 39). Lévi-Strauss discarded chance as an important mechanisms underlying the evolution of societies “Chance certainly exists, but it gives no results by itself”.^2^(our translation) (Lévi-Strauss 1952, p. 59). For Boas and Lévi-Strauss, among others, probability is only a way to control for uncertainty in a deterministic world.

#### Objective probability and the birth of the concept of cultural drift

The objective interpretation of probability (sometimes also called frequentist) claims to the contrary that true randomness exists, i.e. the result given by a roll of a dice can not be predicted, whatever the amount of information available. We are not aware of scholars explicitly adopting this point of view in the field of cultural evolution. However, one can argue that introducing the concept of cultural drift in the study of cultural change was an important step in that direction.

Gulick (1905) was one of the first to argue that stochasticity plays an important role in both organic and cultural evolution, using terms such as the ‘indiscriminate failure or success’ of individuals. The term ‘cultural drift’ was introduced a few decades later (reviewed in Binford 1963 and Cavalli-Sforza and Feldman 1981), but it remained vague and inappropriate: there was a confusion between biased directional changes and changes due to the accumulation of unbiased individual variations. Binford (1963) attempted to define cultural drift in an archaeological framework, using analogies from population genetics, as the product of individual variations inherited through generations and subject to random sampling. He did not believe that cultural drift was strong enough to change ‘norms’, but he thought that it could explain some minor variations observed in the artefacts produced by different groups, and for which no ‘obvious selective rationale’ was at hand. He understood that the analysis of stochastic variations was crucial to the study of the evolution of artefacts, with the potential to produce insights into the structure, organisation and functioning of ancient societies, e.g. revealing an occurrence of exchanges between groups. However, at the time he wrote, formal mathematical tools were not available and thus he could not pursue his research program. Following Binford, Dunnell made significant advances in cultural evolution theory by linking styles of objects in archaeological records to cultural drift ‘because of the independence of style from its environment[…]’ (Dunnell 1978). In his seminal paper, Dunnell explicitly refers to objective probability by employing mathematical terms such as ‘stochastic processes’ or ‘Markov chains’. An equivalent mathematical framework was being developed in parallel by two population geneticists, Cavalli-Sforza and Feldman.

### A mathematical formalisation of the cultural drift concept

**Fig. 1:**
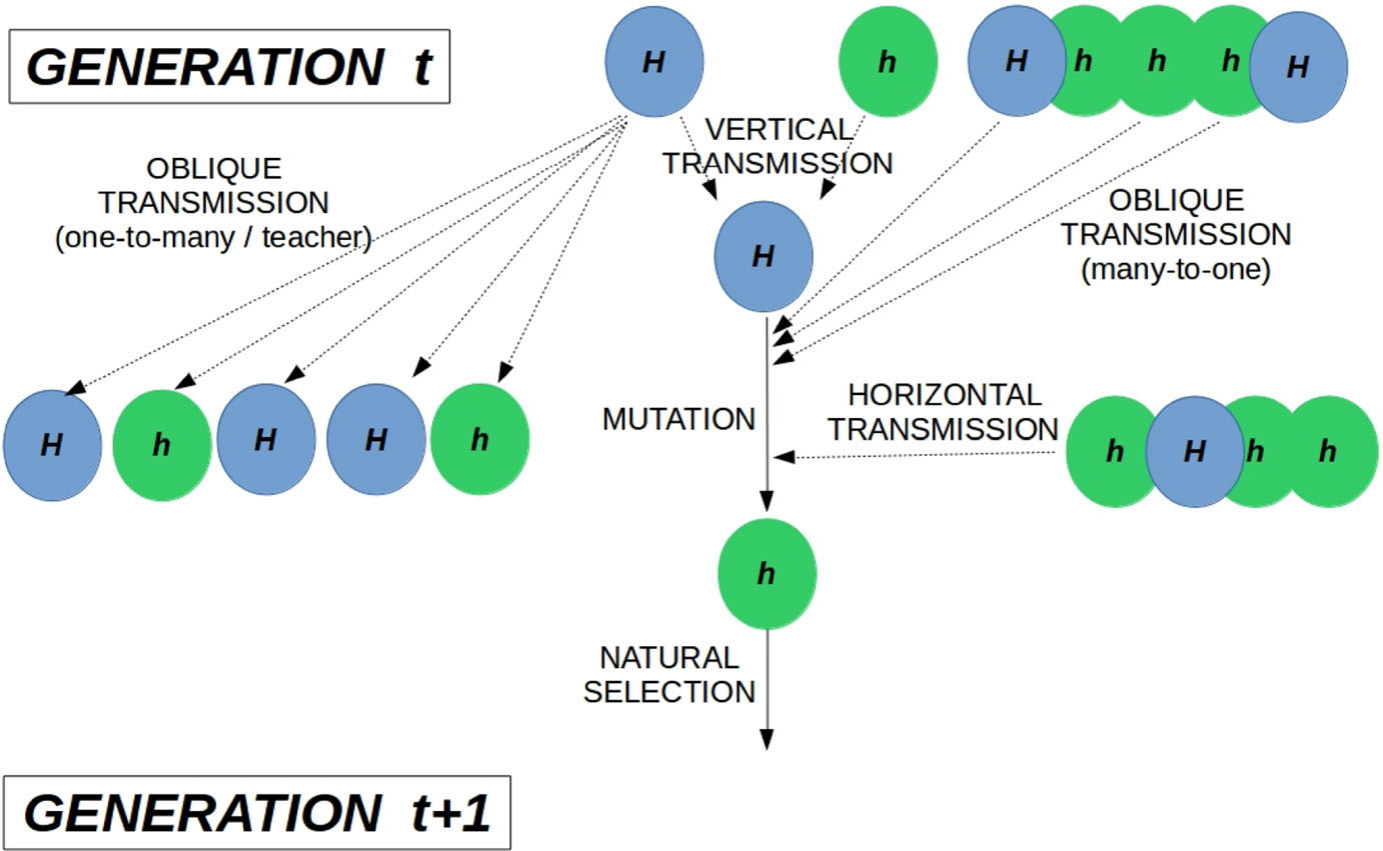
A schematic representation of the stochastic process underpinning cultural evolution in the model of Cavalli-Sforza and Feldman (1981). *H* and *h* represent different cultural traits. Time is discrete, population size is fixed, generations are non-overlapping, mutation occurs at birth and traits are inherited through vertical, horizontal or oblique transmissions. Two social learning routes are depicted: one-to-many and many-to-one. Two types of selection are shown: Darwinian, due to a difference in the rate of reproduction or mortality, and cultural, due to a transmission bias during learning. Biological and socio-cultural processes are assumed to occur in a specific order (birth and vertical transmission, simultaneous oblique and horizontal transmissions, mutation or innovation, natural selection)

The next major step in the definition and utilisation of the concept of cultural drift was taken by Cavalli-Sforza and Feldman (1981). They formalised the dynamics of change of cultural traits (i.e. how the frequency of cultural traits changes in the population over time) by analogy to the stochastic process underlying the dynamics of genetic traits (Fig. 1). Cavalli-Sforza and Feldman (1981, p. 190) considered a standard Wright-Fisher stochastic model, from population genetics, and included some aspects of cultural evolution: they considered the possibility of transmission of cultural traits from parents (vertical transmission) and non-parents (horizontal and oblique transmissions, Fig. 1), with several possible inheritance structures (e.g. one-to-many, teacher to pupils). They considered two causes of selection: natural selection, due to a difference in reproductive or mortality rate (the classic concept of fitness in population genetics), and cultural selection, due to a transmission bias during social interactions. In this framework, cultural drift is modelled through the random sampling of two cultural parents, seen as producing new individuals of the next generation, which is a perfect analogy to how drift is modelled in population genetic models.

Following the work by Kimura and colleagues (see in particular Crow and Kimura 1970), Cavalli-Sforza and Feldman (1981, p. 191) demonstrated that under the assumptions of rare mutations and weak selection, the stochastic process driving cultural evolution can be approximated by the additive effects of two components (“Appendix 1”): one deterministic (natural and cultural selection) and the other stochastic (cultural drift due to random sampling). They demonstrated that cultural drift depends on both (1) the relative strength of vertical, oblique and horizontal transmission, and (2) how learning is organized in the population. Under this framework, it is for example predicted that there should be a positive relationship between the size of a population and its cultural diversity because the effect of random drift is expected to be stronger in smaller population: due to random sampling during cultural transmission, some traits are expected to be lost by chance and with a higher probability when populations are small. Several theoretical studies on cumulative culture do indeed predict that larger populations should exhibit a larger cultural diversity (Lehman et al. 2011; Aoki et al. 2011), which has been tested empirically through both experiments (Derex et al. 2013) and observations (e.g. Kline and Boyd 2010). As a consequence, the occurrence or loss of innovations in archaeological or anthropological data are not necessarily explained by the effect of ecological or economic contexts, which constrain humans to invent, but by the effect of population size: when populations get larger, more diverse and complex cultural traits can coexist, while when they get smaller, technologies can be forgotten. Recently, the “population size” hypothesis for explaining changes in cultural diversity and complexity has been discarded by some scholars for several reasons: the definition of complexity was too vague and arbitrary, and the transmission and learning processes were not compatible with data (Vaesen et al. 2016).

Even if some research focused on the consequences of cultural drift, most of the empirical and theoretical research inspired by Cavalli-Sforza and Feldman (1981) focused on the deterministic part of cultural evolution, i.e. natural and cultural selection (Boyd and Richerson 1985) and cultural attractors (Sperber 1996, note that even if cultural attraction is defined as “statistical” for instance in Claidière et al. 2014, the models are deterministic since they only consider average change over time and not the possible fluctuations of cultural evolution due to chance only). By comparison with the natural sciences, evolutionary biology and ecology (but also chemistry and physics) in particular, stochasticity as a core process in cultural evolution has been little discussed (Cavalli-Sforza and Feldman 1981, p. 309; Mesoudi 2011, p. 76; Lansing and Cox 2011).

### The use of neutral models in cultural evolution: controversies

Even though the idea of using the concept of cultural drift was first proposed over 50 years ago (Binford 1963) and mathematical and statistical methods were available in population genetics since the 1970’s, the first attempt to use neutral models in cultural evolution only occurred in the 1990s in archaeology and in the 2000s in anthropology. As shown below, debates over the importance of stochasticity for cultural evolution have been scarce and limited to the relevance of cultural selection for explaining cultural diversity and change.

#### Neutrality tests in archaeology

Following Dunnell (1978), Neiman (1995) first proposed to apply Ewens’ sampling formula (Ewens 1972) to analyse archaeological data. This method was initially developed for genetic data and it assumed a neutral Wright-Fisher stochastic process. Neiman’s idea was to quantify the diversity of artefacts from a sample and compare it with the diversity expected by Ewens’ sampling formula, according to which the outcome only depends on the sample size. If the observed and expected diversities are similar, then the processes underlying the production of the artefacts are most likely governed by random drift, but if the diversities differ, the processes are most likely underpinned by selection. Neiman (1995), who studied the variations observed in the decoration of ceramics from the Southern Illinois during the Woodland period (200 BC–600 AD), concluded that the diversity observed was mainly due to random cultural drift. Subsequently, Bentley et al. (2004) showed that the variability of pottery decorative motifs on archaeological pottery excavated in western Germany (dated between 5300 BC–4850 BC) was also compatible with neutral expectations, while Shennan and Wilkinson (2001) claimed that the diversity observed in the same sample, but using different statistics, was not captured by a neutral model. They interpreted this result as evidence for a selection bias in favour of novelty (rare variants are preferred) in the later phase of the period studied.

However, Neiman (1995), Bentley et al. (2004) and Shennan and Wilkinson (2001) did not test whether the differences between the observed and expected diversities were statistically significant. This limit was later overcome by Shennan and colleagues, still on the basis of a neutral Wright-Fisher model. Kandler and Shennan (2013) developed a method for predicting the expected change in the frequency of cultural traits through time, and they derived an approximation in order to detect departures from neutrality. They applied their method to the same data set as Shennan and Wilkinson’s (2001), and they confirmed that the neutrality hypothesis had to be rejected. Another move forward was contributed by Crema et al. (2014), who developed a comparative approach to evaluate the fit of several competing models to the data (Burnham and Anderson 2002), an improvement from assessing the fit of a null model only.

#### Neutrality tests in anthropology

In anthropology, there has recently been a strong impulse to find neutral cultural traits that could be used to test the importance of cultural drift versus cultural selection for driving cultural evolution. Bentley and colleagues started to analyse the patterns and dynamics of the frequency distribution of dog breeds (Herzog et al. 2004), song charts (Bentley et al. 2007), scientific keywords in papers (Bentley 2008), and first names given at birth (Hahn and Bentley 2003; Bentley et al. 2004). The goal behind those studies was to simulate a neutral Wright-Fisher model for generating distributions of frequencies, turn-over rates and other statistics, which could then be compared to the observed patterns of different cultural traits. The main conclusion of this research is that cultural drift is indeed an important process, but the possible role of cultural selection is not excluded.

In the 2000s and 2010s, several papers challenged these conclusions (e.g. Richerson and Boyd 2008) arguing that when looking at other statistics, the neutral theory is not compatible with observations, which would support the idea that several processes other than cultural drift can generate probability distributions identical to those obtained from neutral cultural evolution models. For instance, the dynamics of change of the frequencies of first names and dog breeds show a common feature that is not expected from a process of random drift: the rates of adoption and abandonment are positively correlated, i.e. the faster first names or dog breeds reach high frequencies, the faster they disappear afterwards. In other words, the more popular a name or a dog breed is, the faster it is abandoned after having reached a high frequency (Berger and Le Mens 2009; Acerbi et al. 2012; Kessler et al. 2012). Berger and Le Mens (2009) argued that this is due to psychological or social biases against short lived cultural items. Kessler et al. (2012) and Acerbi et al. (2012) explained the positive correlation by a memory effect and concluded that simple rules of cultural transmission could explain patterns at the population level, rejecting the neutral model used by Bentley and followers.

## Stochasticity in cultural evolution: the reasons for a lack of controversy

In the cultural evolution literature, both deterministic and stochastic positions do exist. On the one hand, a deterministic view is adopted by most archaeologists and anthropologists (see for instance quotes by Boas and Lévi-Strauss before). Braun (1992) explained that “most archeologists shy away from explaining at least most cultural changes as random”. On the other hand, scholars like Dunnell (1978), Cavalli-Sforza and Feldman (1981) and their followers assume that stochasticity plays a central role in explaining cultural change. However, the intensity of the opposition between defenders of each view is strikingly different between ecologists and evolutionary biologists (and most other sciences of nature) and cultural evolutionists. In population genetics and ecology, the neutral theory has fundamentally challenged research paradigms and programs (see “Appendix 2” and references therein for a quick overview). In cultural evolution, such a ‘stochastic revolution’ has yet to happen despite the fact that cultural drift has been proposed to be a fundamental causal process for the evolution of cultural traits for over three decades (Dunnell 1978; Cavalli-Sforza and Feldman 1981) if not longer (Binford 1963). We shall now speculate about the reasons why that should be so.

### A general aversion to evolutionary and quantitative approaches to culture in anthropology

The first evolutionary approach to the study of culture was developed in the 19th century by two anthropologists—E.B. Tylor and H.L. Morgan—who infamously came up with a model of unilinear cultural evolution according to which societies could be ranked according to their levels of complexity or “progress” (Tylor 1871; Morgan 1877). This approach came to be known as “evolutionism”, which helped justify social Darwinism and colonization, and which was later proven to be theoretically and empirically incorrect. After evolutionism fell into disrepute, and after anthropology moved on to adopt Franz Boas’ cultural relativism, cultural evolution enjoyed a renewed interest through the development of cultural ecology, an approach pioneered by J. Steward in the second half of the twentieth century. For Steward, cultural groups were not only divergent, they could go on parallels tracks, due to cross-cultural phenomena resulting from environmental similarities (Steward 1990). He saw technology and environments as the main causes of cultural evolution, and culture as an adaptation to the environment. This approach, later broadened into materialist anthropology, somehow declined in mainstream anthropology after having been criticized by the structuralists for focusing on material conditions rather than thoughts, by the postmodernists for using the scientific method, and by the relativists for using cross-cultural comparisons. Ultimately, mainstream social anthropology abandoned any evolutionary approach to the study of human culture, questions about the relative role of stochasticity versus selection for explaining cultural evolution were thus not relevant.

There is also an anthropological aversion for quantitative approaches (e.g. Ingold 2007). This aversion has had important consequences for the study of cultural evolution since it led to a dramatic lack of quantitative predictions. A common feature between population genetics and community ecology was the clear discrepancy between theoretical quantitative predictions and empirical observations, which motivated the development of stochastic models. Population geneticists were not able to explain the huge diversity observed at the molecular level with selectionist models only, and community ecology could not explain the large number of species co-occurring in a locality with deterministic models alone. In both disciplines, quantitative predictions were available for decades thanks to the use of mathematical models. In the field of cultural evolution, however, there was no quantitative framework for generating predictions of patterns of cultural diversity until the 1980s. Concluding to problematic discrepancies between theory and data was thus not possible, precluding the need for an assessment of several alternative theories to explain cultural change and diversity.

### Debates are elsewhere

For most scholars in the human sciences, the question of whether patterns are best explained by stochastic or deterministic processes is not central and there is no opposition between two alternative theories. Other questions are the focus of attention: To what extent do individual interest and group values drive cultural changes? To what extent are human practices constrained by power structures? What is the impact of conscientiousness and individual free-will on patterns of cultural diversity? Internal divisions within anthropology are numerous and long-standing, especially between social/cultural anthropology on the one hand, and physical/biological anthropology on the other hand, and the ‘anthropological project’ is certainly not well defined (Ingold 2007). In short, from the beginning of the twentieth century, there was no favourable ground for the emergence of a controversy over the role of chance in cultural evolution.

In the specific context of cultural anthropology, the most important and violent debate concerns whether it is legitimate or not to adopt an evolutionary approach for understanding cultural diversity at all (e.g. Mesoudi et al. 2006; Ingold 2007, 2013). For opponents of the evolutionary approach, it is perhaps not surprising that the role of cultural drift is not discussed since evolutionary theory is rejected as a whole. However, more surprisingly, even for scholars supporting an evolutionary approach in cultural evolution, debates mostly concern the mechanisms underlying selection, and whether or not natural and cultural selection are analogous (Claidière et al. 2014; Acerbi and Mesoudi 2015). The research questions, which led to vigorous debates, mainly engaged with the definition of cultural selection: Is it Darwinian or not? How and why is cultural transmission biased? How does culture coevolve with genes? It could be argued that it would first be necessary to establish consensus for the definition and importance of cultural selection if it is to be opposed to cultural stochasticity. Yet, it does not explain why the evolutionary debate started with the concept of cultural selection, as opposed to that of cultural stochasticity, given both selection and stochasticity are equally important processes in the neo-Darwinian theory of evolution.

### Chance as a necessity against determinism and in defence of free will

In social anthropology and other human sciences, there is resistance to a deterministic perspective: free will is, for most scholars, fundamental constituents of human beings and societies. Determinism and free will are taken as incompatible. Since at least the nineteenth century, some social scientists have seen stochasticity as a means to defend individuality and freedom (Gigerenzer et al. 1989, chap. 2), justified by different interpretations of probability. On the one hand, a subjective interpretation of probability has been used to explain the differential evolution of individuals and civilizations by Boas (1938) and Lévi-Strauss (1952). What they had in mind is a limited knowledge of the real causes of events: there are so many interactions between individuals, societies and their environment that knowledge of causality is out of reach, especially when it comes to human creativity and cultural innovation. This warrants the use of statistics and probabilistic methods to describe the diversity of cultural traits (Hacking 1990). Adopting a subjective interpretation of probability can also explain that observed variations are biased and relative to the observer. On the other hand, all the way back to Charles Bernard Renouvier in the 19^th^ century (Gigerenzer et al. 1989, p. 64–65), some scholars have adopted an objective interpretation of probability as necessary to the human condition and allowing for free will (e.g. Provine 2014). Finally, some partisans of cultural drift ratonalize the use of stochastic models in part by the fact that each individual might have its own reason to prefer a cultural variant over another (e.g. Kandler and Shennan 2013), thus mixing subjective and objective interpretations of probability. Stochasticity thus appears as an adhoc solution to the ‘problems’ of free will and individuality, obliterating the need for a controversy. The adoption of a very general and vague concept of chance in the human sciences hides multiple interpretations, each scholar having their own conception of chance.

### Cultural drift as a part of a whole

A possible explanation for the rarity of controversies over the role of stochasticity in cultural evolution is that cultural drift is a component of a coherent paradigm: the neo-Darwinian evolutionary theory. In most of the cultural evolution literature, which borrows theory and methods from evolutionary biology, cultural drift is presented as an evolutionary force along with migration, mutation and selection (Acerbi and Mesoudi 2015), and this is also true of opponents of evolutionary archaeology (e.g. Boone and Smith 1998) or evolutionary anthropology (e.g. Palsson 2013). Applying an evolutionary approach that considers both deterministic and stochastic processes is an explicit claim, for instance in Lipo and Madsen (2001): “From the beginning of archaeological efforts to incorporate evolutionary theory into practice, both neutral and adaptively significant variation have been important to theory-building efforts”. Taking a classical image from population genetics, one can argue that ‘cultural drift’ is being hitch-hiked in the population of scholars supporting an evolutionary approach in cultural evolution studies: it invades this scientific community not because it is successful in itself but because it is associated with other successful cultural traits (Hurt et al. 2001).

The absence of debates over the role of chance in cultural evolution studies might be partly due to this hitch-hiking effect: cultural drift is a part of the whole of the neo-Darwinian theory, if one adopts the latter, one must adopts the former as it is. Indeed, the assumption is that the question of neutrality is settled in evolutionary biology, and thus understanding the relative role of stochastic versus deterministic processes does not warrant further research attention. However, importing the theoretical framework from evolutionary biology to cultural evolution can have drawbacks, and stochasticity may be compared to a Trojan horse: whenever there is an interdisciplinary migration of techniques or concepts, it comes with a pack of assumptions (Gigerenzer et al. 1989, p. 273). In the last part of this paper, we expose a critical analysis of the analogy between cultural and biological drift.

## Limits of the analogy between biological and cultural drift: beyond Wright-Fisher

The stochastic processes in population genetics and community ecology models are the Wright-Fisher’s and the Moran’s models with immigration, respectively (“Appendix 1”). It is important to note that these stochastic processes have been chosen by population geneticists and ecologists according to their empirical questions. In population genetics, the reference model corresponds to a species with non-overlapping generations, with a single simultaneous event of reproduction for all individuals and with no population structure. This model is chosen because it is encountered in nature (think about annual plants or fruitflies). In community ecology, Hubbell chose the Moran’s model with immigration because it corresponded to his knowledge of the functioning of communities of tropical trees. By contrast, there is no empirical rationale for choosing the stochastic process of reference in cultural evolution models. To date, almost all models (see some exceptions below) adopted the framework of Cavalli-Sforza and Feldman (1981), i.e. the Wright-Fisher model used in population genetics (Fig. 1, e.g. Bentley et al. 2004; Mesoudi and Lycett 2009; Rorabaugh 2014). This choice is questionable, however, and another stochastic process could provide a better reference. Below we list a number of assumptions taken from the Wright-Fisher model and discuss their validity for modelling cultural drift.

In this section, we contend that cultural processes should be modelled differently from genetic processes. There are sources of stochasticity that are specific to culture and which might be revealed by asking the following questions: How is information expressed and transmitted? How do individuals make decisions? How do individuals interact? How do cultural traits affect demographic and interactions processes? From the individual to interacting individuals to the population, several mechanisms at different levels of organization can produce stochasticity. We conclude that cultural evolutionists should develop a stochastic theoretical framework that considers culture-specific sources of stochasticity, which will facilitate the interpretation of the variability observed in archaeological and anthropological data.

### Modes of interaction and information transmission

A direct interaction between two or more individuals can be necessary for the transmission of cultural traits, and how individuals interact is variable. The supposed structure of the interaction network in cultural evolution matters because it dramatically impacts the dynamics of the selected traits (Nowak 2006) and the extent of the diversity maintained in a population (Cavalli-Sforza and Feldman 1981; Powell et al. 2009; Tan et al. 2015). In the case of cultural evolution, there are undoubtedly too many possible population structures for producing a general model. However, it is not clear that the basic assumption should be a complete network, such as in the Wright-Fisher’s model, or any other random social networks.

Another limit of current cultural evolution models is that cultural transmission is assumed to be unilateral: there is a donor and a receiver. Although such assumption is reasonable for genetic transmission, its validity is less clear for cultural transmission. One can argue that cultural transmission is generally bilateral: individuals are both donors and receivers. For instance, a teacher can transmit knowledge to a pupil, and the latter’s reaction to the teaching can change the knowledge of the teacher. Experiments show that information can indeed be bilaterally transmitted in animals: fruitflies can be trained to prefer a medium on which to lay eggs, and can transmit that behaviour to naive individuals. Naive individuals can also transmit their ‘ignorance’ to trained individuals (Battesti et al. 2015). Billiard et al. (2016) theoretically demonstrated that the bilateral transmission of traits can have a dramatic impact on the maintenance of diversity in a population, the size of the stochastic fluctuations and the probability and time of fixation of traits.

Finally, in models of cultural evolution, it is only an expressed trait that can be transmitted, whereas in genetic evolution, the whole genome is transmitted at each reproductive event, independently of the expression of genes. This point has been highlighted by some authors (Mesoudi 2011), but to our knowledge, there has been no debate and therefore no consensus has emerged on how this should be treated.

The evolution and impact of different cultural selection biases (such as conformist-bias or prestige-bias) on the dynamics of cultural evolution has attracted much attention (e.g. Boyd and Richerson 1985; Hoppitt and Laland 2013; Voinson et al. 2015). Cultural selection biases are sometimes discussed as “cognitive biases” in the sense that individuals choose a cultural variant because of their evolved cognitive dispositions. While cultural selection biases have some empirical justifications (Mesoudi 2011), they are a priori hypotheses about biased transmission of cultural variants at the level of the population, and they do not emerge from the interaction of individuals (in contrast to the methodological framework developed by Cavalli-Sforza and Feldman 1981). We believe that this is a particularly important challenge for the future of cultural evolution modelling. The next step is to explicitly model the transmission of cultural traits at the individual level and show how it translates into cultural selection at the population level.

### Biases due to random copy error

It is generally assumed that mutation and innovation do not introduce biases per se but only through their effects on transmission, reproduction or mortality. Similarly, copy error during transmission is generally thought to be bias-free. However this is not generally true. For instance, in the psychological literature, it is well-known that the perceptive abilities of humans are constrained by their physiology. Copy errors are relative to the size of the artefact: the Weber Fraction posits that an individual is unable to detect a difference within a 3% range (Eerkens and Bettinger 2001). Following Eerkens and Lipo (2005), Hamilton and Buchanan (2009) developed a stochastic model for the dynamics of cultural traits explicitly accounting for the Weber Fraction limit. They assumed an error normally distributed with a mean of zero and a variance proportional to the size of the copied artefact, but without specifying an explicit transmission process between individuals. In this model, cultural drift is not present because individuals are not randomly sampled during cultural transmission. Hamilton and Buchanan (2009) showed that the stochastic process generates a negative bias, i.e. the artefact is expected to evolve towards a smaller size: since errors are proportional to the size of the artefact, the most accurately transmitted artefacts are the smallest. This example illustrates that cultural evolution can be generated by processes altogether different from cultural selection, in this instance individual copy errors, which are different from cultural drift (due to random sampling error during cultural transmission).

### Time scales

An implicit assumption underlying the Cavalli-Sforza and Feldman (1981)’s model is that all processes (birth, vertical cultural transmission, horizontal and oblique cultural transmission, mutation and natural selection) play out on similar time scales, which is debatable. First, interactions between individuals and cultural transmission should generally occur at a much faster rate than death and birth. Second, concerning the innovation rate, the situation differs from population genetics or community ecology where it is generally assumed that mutation or speciation (i.e. innovation) are rarer than birth and death. In cultural evolution, not only the innovation rate can be much faster, but variability can be generated during each transmission event because cultural transmission is often imperfect. An individual can also change its state by its own experience (individual learning, Hoppitt and Laland 2013), and innovations can also occur without social interactions. Third, the cognitive sciences show that making decisions is a stochastic process that takes some time (Forstmann et al. 2016).

Three levels of analysis should be considered simultaneously for the evolution of culture: the individual level (how the trait is modified during transmission, individual experience and decision-making), the level of social networks (who interacts with whom, what is transmitted), and the population level (birth and death). The relative time scales between the three levels must be explicitly specified. Each level can be a source of stochasticity and may affect the dynamics of trait evolution, even though models generally only consider one level. This is also true of population genetics, where, in most cases, it is only stochasticity from random sampling during both reproduction and the transmission of genetic information that is considered, while there are other sources of stochasticity due to mutation or the environment (Lenormand et al. 2009). Thus far, in all models of cultural evolution (but see Eerkens and Lipo 2005 and followers), stochasticity only comes from cultural drift, i.e. from the random sampling of individuals during cultural transmission. Whether random sampling is the most important source of stochasticity in cultural evolution, as compared with innovation or decision-making, for instance, remains an open question.

### Population size

In population genetics, community ecology and cultural evolution models, population size is generally assumed to be constant and independent of the traits, i.e. when a selected trait invades a population, be it favourable or deleterious, the population size is not impacted even if selection changes the birth rate or the death rate of individuals. However, some population genetics models demonstrated that a feedback between selection and population size can have a considerable effect on evolutionary dynamics, leading populations to extinction (Lynch et al. 1995; Abu Awad et al. 2014). The hypothesis of independence between a selected trait and population size can be justified in population genetics and ecology because one can assume that the environment is at its carrying capacity. This rationale is questionable for modelling cultural evolution, however, especially in the context of technological innovations directly influencing population size, for instance agriculture, birth control or medicine.

Population size can vary with the adoption of cultural traits. For instance, contraceptive use can be considered as a cultural trait, and cultural evolution studies have researched the necessary conditions for demographic transitions to occur (e.g. Fogarty et al. 2013). Since contraceptive use might have a direct effect on fertility, population size may change in response to changes in contraceptive behaviour (e.g. Alvergne and Billiard in preparation). More generally, the dependence of population size on cultural traits can make us reconsider the interpretation of the observed positive correlation between diversity, the complexity of technologies and population sizes (e.g. Kline and Boyd 2010; Vaesen et al. 2016). This positive correlation is usually interpreted as evidence for the effect of cultural drift by evolutionary scholars. Yet, this correlation can be interpreted the other way around: the more complex and diverse the cultural traits, the larger the population. Other indirect effects have also been proposed to explain the relationship between cultural complexity and population size (Vaesen et al. 2016): for instance, larger populations can favour the emergence of highly specialized individuals, using more complex skills and tools. The ongoing debate in archaeological studies to explain the relationships between population size and complexity is a fertile ground on which to build a stochastic framework for cultural evolution.

### Time assemblages and fluctuations in the conservation of archaeological data

The structure of cultural data can be thoroughly different from the structure of biological data. In archaeology, a sample is spatially well defined but it often covers a large timespan. Thus, archaeological data are an assemblage of artefacts, produced by individuals who possibly never interacted (Neiman 1995). Premo (2014) showed through computer simulations that assemblages can generate biases that do not result from specific transmission patterns between individuals. Indeed, time assemblages can generate a large diversity of cultural traits because of the merging of different time strata. A diversity larger than expected from neutrality can thus arise from methodological caveats and the assemblage itself rather than from specific transmission biases. Such overlap between different time strata is probably inherent to most archaeological data. A promising statistical framework taking into account the specificity of archaeological data is currently being developed (Kandler and Shennan 2013; Crema et al. 2014, 2016). However, several questions remain, for instance that of the role of stochasticity in the conservation of artefacts through time. Stochastic fluctuations in conservation can be so important that other sources of stochasticity would be negligible.

## Summary and conclusions

In this article we have reviewed the studies that have engaged with the importance of stochasticity, i.e. chance, in cultural evolution. Drawing on research published in archaeology and anthropology, we focused on the development and use of a ‘neutralist’ theory (i.e. when change is not biased towards one particular direction). We found that in contrast with the stochastic turn that has occurred in population genetics and community ecology, controversies over the importance of stochasticity in cultural evolution have remained scant. Ironically, it is the social sciences that have triggered the stochastic revolution in the natural sciences, even reaching physics (Gigerenzer et al. 1989). Indeed, the field of statistics has been developed since the beginning of the nineteenth century, mainly because of the accumulation of data from social studies, to explain and interpret inter-individual variation. While sociology is now at the forefront of the development of statistical methods for analysing social interactions (e.g. Snijders 2017), in social and cultural anthropology, since the interpretive turn initiated by Geertz and Evans-Pritchard, quantitative approaches to society and culture fell into disrepute.

The general conclusion of our paper is that the question of which fundamental stochastic processes underlie the evolution of cultural traits deserves more attention and critical thinking than it currently receives. While there is now a relative consensus over the form of the most basic stochastic models in population genetics and community ecology, the issue has hardly been raised in the field of cultural evolution. Indeed, following Cavalli-Sforza and Feldman (1981), in analogy with population genetics, cultural drift is defined as the effect of random sampling of cultural parents between generations. However, there are several other potential sources of stochasticity that might impact patterns of cultural diversity: cognitive processes at the individual level, random copy errors during the expression of traits, interactions between individuals in social networks, and random sampling at the population level. Questioning the fundamental differences between cultural and genetic drift might be as important as debating the correspondence between cultural and natural selection for assessing how valid the analogy between cultural and biological evolution is.

There might not be a single answer to the question of which stochastic processes underpin cultural evolution, but working towards answering that question might both facilitate the development of methodologies better suited to the analysis of cultural data and clarify the research program pursued by cultural evolutionists. For instance, the variability observed in cultural data is usually taken as the manifestation of statistical noise. However, this noise can contribute information that ought not to be neglected since it directly arises from the transmission of cultural traits and from demographic and psychological processes, i.e. from the stochastic processes underpinning cultural evolution (see Sect. 4 and “Appendix 1”). In addition, while it is possible to contrast the empirical diversity of cultural traits with predictions from different stochastic models, directly applying methods from population genetics to the analysis of cultural data might produce caveats because of the particularity of the stochastic processes involved in cultural evolution. For instance, different cultural traits do not necessarily share the same demographic and transmission histories, and they might transmit along different routes (e.g. some traits are transmitted from parents, others from peers, teachers or friends; see Boyd et al. 1997 for further discussion). One of the biggest challenges for the development of stochastic models in cultural evolution is to work out how to use the variability of cultural traits as a proxy for inferring the demography or history of populations, disentangling all sources of variability from the level of the individual to that of the population.

Finally, we focused our paper on studies conducted by scholars using an evolutionary and quantitative approach. For cultural evolutionists, it is clear that culture evolves because of processes analogous to those involved in biological evolution, including random drift. However, the opponents of an evolutionary approach to culture argue that other processes underlie cultural diversity. For instance, Ingold (2004, 2007, 2013) believes that the transmission of cultural traits between individuals is only a metaphor (Ingold 2013, p. 15). He accounts for cultural diversity by invoking the constant development of individuals as self-organizing entities. In this view, what individuals know and think and how they behave is due to constant feedback interactions between their development and their ecological and social environments. The idea that cultural diversity has evolved from the self-organizing properties of humans rather than some kind of inheritance mechanism aligns itself well with theories that some biologists have proposed as alternatives to the neo-Darwinian paradigm in biology (De Tiège et al. 2015). Yet, similarly to followers of Cavalli-Sforza and Feldman (1981), the relevance of the analogy between biological and cultural processess is not discussed in the light of the role of stochasticity. Why that is the case remains an open question.

## Appendix 1: Stochastic processes and neutral models

### Population changes as stochastic processes

Imagine we are interested in how a variable *X*_*t*_ changes with time *t*. This variable can be the frequency of genetic variants (in population genetics), the size of a population (in ecology) or the attribute of artefacts in archaeological assemblages. If the change of *X*_*t*_ with time is stochastic, the sequence of the values taken by *X*_*t*_ is called a stochastic process. The change of *X*_*t*_ can depend on different mechanisms (e.g. innovation, random sampling, selection, transmission or copy error), which can be summarized in the function *F* as

1 $$X_{t + 1} - X_{t} = F(t,X_{t} )$$

In population genetics, the Wright-Fisher model consists in producing offspring at time *t* + 1 by randomly sampling their parents from the population at time *t*. *F* is then given by a multinomial distribution of which the parameters are the frequencies of the different alleles in the population at time *t*, weighted by selection and mutation rates. Most models of cultural evolution also assume a multinomial distribution for *F*. In community ecology, the Hubbell’s model is slightly different since it consists in exchanging a single uniformly chosen individual at each time step (a so-called Moran’s model) with the offspring of another individual from within or outside the local community.

### Approximation of the stochastic process: deterministic versus stochastic parts

Under appropriate assumptions about the scalings of the different parameters, stochastic processes can generally be approximated by (Ethier and Kurtz 1986)

2 $$X_{t + 1} - X_{t} = \mu (t,X_{t} ) + W(t,X_{t} ).$$

The first term in Eq. (2) *μ*(*t*, *X*_*t*_) = *E*[*F*(*t*, *X*)] is the expectation of the function *F* and is called, in mathematics or physics, the deterministic or drift part of the stochastic process (not to be confused with the term genetic drift or cultural drift). The average direction followed by *X*_*t*_ is modelled by the deterministic part. Selection tends to bias the direction of evolution and is thus a component of the deterministic part *μ*(*t*, *X*_*t*_) (Cavalli-Sforza and Feldman 1981). Mutation, innovation, copy-error or migration can also produce biases.

The second term in Eq. (2) is generally called the stochastic part of the stochastic process with *W*(*t*, *X*_*t*_) a random variable giving the fluctuations around the deterministic evolution of the population. Often, *W*(*t*, *X*_*t*_) follows a centred Normal distribution with variance *σ* ^2^(*t*, *X*_*t*_) but it can have other components, such as random jumps when large effect mutations or environmental catastrophes occur. In population genetics, the stochastic part *W*(*t*, *X*_*t*_) is generally assimilated to genetic drift (fluctuations of the genetic frequencies due to random sampling), and to demographic stochasticity in ecology (fluctuations of the size of a population due to random birth and death). The variance *σ* ^2^(*t*, *X*_*t*_) of *W*(*t*, *X*_*t*_) is generally dependent on the size and the structure of the population. Large populations are characterized by a low variance, i.e. fluctuations around the expectation of the stochastic process are small. The structure of the population in classes, subpopulations, sexes, etc. can modulate this effect, which is captured by the concept of effective population size in population genetics.

### A definition of neutral models

Neutral models can be defined as special cases of Eq. (2) where there is no bias, i.e. *μ*(*t*, *X*_*t*_) = 0. In population genetics, it generally corresponds to assuming that the fitness of individuals does not depend on their genotypes, and in community ecology that migration, birth and the death rates of individuals do not depend on their species. In models of cultural evolution, neutral models assume that the mortality and reproduction of individuals are not affected by their cultural traits, but also that all cultural traits have the same propensity to be transmitted between individuals. In population genetics, community ecology and cultural evolution, all neutral models also assume that the process generating diversity (mutation, speciation or innovation) does not introduce inherent biases.

## Appendix 2: Contribution of ‘neutral’ models in ecology and evolution: A quick overview

### Population genetics and evolutionary biology

Population genetics models were developed during the 1920s–1930s, mainly by R. A. Fisher, S. G. Wright and J.B.S Haldane (Provine 1971). Their goal was to provide a mathematical framework for studying how biological evolution unfolds given Darwinian natural selection and Mendelian genetic inheritance. Very rapidly, Fisher highlighted that stochasticity should affect evolutionary dynamics because populations are finite. Fisher’s view was later confirmed and refined by Haldane and Wright (Provine 2014). However, Wright and Fisher thoroughly disagreed with regards to the importance of stochasticity in evolution, which they coined ‘genetic drift’. Fisher claimed that the importance of ‘genetic drift’ was negligible relative to that of natural selection in the long run, while Wright thought that genetic drift was fundamental for the adaptation of species. Despite this debate, the consensus was that the most important evolutionary force for biological evolution was natural selection. Consequently, any phenotypic variation observed within or between species was systematically interpreted as the consequence of natural selection. In the 1960s, Kimura (1968) and King and Jukes (1969) developed the so-called ‘neutral theory of molecular evolution’, arguing that most observed variations were due to neutral or nearly-neutral mutations, i.e. mutations which do not or only slightly affect the reproduction and survival propensities of individuals (Kimura 1983). A violent controversy ensued, opposing the “neutralists” against the “selectionnists”. The debate lasted until the advent of molecular biology, which revealed that a large amount of polymorphism at the molecular level (mostly proteins, DNA and RNA) was poorly explained by selection alone, except in the context of complex assumptions that were not supported by the data, for instance the ubiquitous existence of over-dominance to explain high frequencies of heterozygotes (Kimura 1983). After this controversy, the importance of drift in evolution became widely accepted by population geneticists and evolutionary biologists alike. ‘Neutral models’ of evolution, i.e. where only ‘genetic drift’ occurs, even reached the status of ‘null models’: in order to show that selection does indeed explain an observed pattern, one must show that it deviates from what would be predicted by a ‘neutral model’ (Kimura 1983).

### Ecology

Population and community ecology aim to explain the often considerable species diversity observed at different scales. The models used for studying the conditions for the coexistence of species date back to the deterministic models of Lotka and Volterra and the concept of ‘ecological niche’, i.e. the set of environmental conditions allowing the existence of a species, including the interaction between species (Chesson 2000). The overall picture that emerged from those models is that the range of conditions for which the coexistence of a large number of species is predicted is narrow, which contradicts what is observed in natural populations. Thus, while until then demographic stochasticity was expected to play a minor role in the diversity of communities (Schaffer 1981), this expectation changed in the 1990s, when S.P. Hubbell developed a stochastic framework for community ecology inspired by population genetics (Hubbell 2001). He assumed that all species of a given community were ecologically equivalent and that at each time step a randomly chosen individual dies and is replaced by an offspring from another individual. This constituted the so-called ‘neutral theory of biodiversity’. Hubbell showed that a large diversity of species could be expected due to the balance between the loss of a species resulting from demographic stochasticity and the gain of new species due to immigration from a regional pool. Similarly to what occurred in population genetics, these stochastic models led to a violent controversy which opposed the defenders of the ecological niche theory against those of the neutralist theory (Pocheville 2015). The controversy in ecology is much more recent than in population genetics, however a consensus seems to emerge: there are too many arguments against the neutral theory in ecology to accept demographic stochasticity as a major predictor of diversity. For instance, given that both the ecological interactions between species and the neutral theory generate, under certain scenarios, similar predictions, neutral models can not be used as null models. In addition, contrarily to over-dominant selection which had poor empirical support in population genetics (Kimura 1983), interactions between species are ubiquitous in natural populations.

## Footnotes

1 “On en arriverait donc, pour expliquer les différences dans le cours des civilisations, à invoquer des ensembles de causes si complexes et si discontinus qu’ils seraient inconnaissables […] Cette situation justifie l’introduction dans les sciences sociales de la notion de probabilité”.

2 “Le hasard existe sans doute, mais ne donne par lui-même aucun résultat”.
